# Impact of Individual, Familial and Parental Factors on Adolescent Smoking in Turkey

**DOI:** 10.3390/ijerph18073740

**Published:** 2021-04-02

**Authors:** Coskun Oztekin, Mehak Batra, Shady Abdelsalam, Tijen Sengezer, Adem Ozkara, Bircan Erbas

**Affiliations:** 1 Department of Family Medicine, School of Medicine, Hitit University, Corum 19000, Turkey; coskunoztekin@gmail.com; 2 Department of Public Health, School of Psychology and Public Health, La Trobe University, Melbourne 3083, Australia; mehakcd@gmail.com (M.B.); 20139361@students.latrobe.edu.au (S.A.); 3 Department of Family Medicine, Ankara Numune Research and Training Hospital, Ankara 06000, Turkey; tijensengezer@hotmail.com (T.S.); ademozkara@yahoo.com (A.O.); 4 Faculty of Public Health, Universitas AirLangga, Surabaya 60132, Indonesia

**Keywords:** adolescent, risk, smoking, parental attitudes, parental behaviours

## Abstract

The burden of adolescent cigarette smoking is substantial. We assess mothers’ and fathers’ attitudes and behaviours on adolescent smoking using a cross-sectional study of *n* = 707 adolescents. Associations between parental attitudes and behaviours in adolescent smoking were assessed using logistic regression separately for boys and girls. Occasional alcohol use by both parents increased odds of smoking once a day (OR = 2.44, 95% CI 1.26, 4.71, OR = 1.51, 95% CI 0.97, 2.35, respectively). Fathers smoking increased odds for girls (OR = 1.59, 95% CI 1.01, 2.52). A democratic mother decreased odds for boys (OR = 0.32, 95% CI 0.10, 0.93) whereas a protective, demanding mother increased the odds for girls (OR = 8.65, 95% CI 1.38, 54.22). Public health smoking prevention programs could support changing parental behaviours and attitudes in early years to address this burden in countries with authoritarian parenting styles.

## 1. Introduction

Cigarette smoking kills almost 8 million people each year and remains the number one preventable cause of death [1]. From 2000–2017, globally, around 24.1 million children aged 13–14 years (about 7%) smoked cigarettes and they still continue to do so. Many factors such as biological, psychosocial, and environmental contribute to these behaviours. For example, poor performance in studies, anxiousness, habitual consumption of alcohol, smoking behaviours of parents, siblings, and especially friends, play a role as risk factors [2] in raising the possibility of starting smoking and the increased likelihood of continuing. The home environment does seem important, though. Parental non-smoking, and family monitoring and bond [3] seem to be protective, which may contribute to lowering the likelihood of smoking initiation, as well as reducing the likelihood of continuing smoking. The synergistic relationships between the risk and protective factors contribute to the individual’s overall risk profile. To intervene with smoking behaviour, it is imperative to understand the role these factors play to develop early interventions accordingly.

Most European countries have reached great milestones in terms of curbing the tobacco epidemic. However, challenges are still present. Compared to many other countries, tobacco usage remains high in countries like Turkey. Although prohibition has been implemented for smoking advertisements, promotion, and sponsorship, populations with varying demographics continue to smoke. Moreover, a substantial number remain exposed to tobacco smoke at home or in private cars as the Tobacco Control Law does not cover private premises [4]. Countries like Turkey with 30% of its total population being under 15 years of age are faced with a continual burden of smoking uptake at young ages [5]. Resistance among adolescents to interventions that prevent them from smoking is also a further challenge which is a common phenomenon irrespective of country of origin.

Studies [6,7] have assessed parenting behaviours on smoking in adolescents as one way to better understand early uptake of childhood risk-taking behaviours. However, these studies are lacking in countries such as Turkey where smoking in younger populations is prevalent [8,9]. Studies have either examined the parenting styles of the mother or father alone but not in combination and they haven’t included smoking and alcohol behaviours of the parents when assessing these effects. Further, across all regions except the Eastern Mediterranean region, the rates of smoking among boys are consistently about 9–10%. For girls, the prevalence seems to be higher in high-income countries whereas for boys the higher prevalence rate is in the upper-middle-income countries such as Turkey [10]. Quitting is always difficult and least common in adolescents. Girls are often faced with many barriers to smoking cessation and are at increased risk of smoking-related mortality and morbidity [11]. Their peers and their standing amongst them both impact their uptake and continuation of smoking [12]. Compared to their male counterparts, younger girls take up smoking as it is deeply related to the vigour of self-concept, i.e., how they identify their appearance and physicality in relation to others [13]. Parental attitudes and behaviours may be important but their role in smoking may be different for boys and girls.

Utilising a large cross-sectional study conducted in the Ankara province of Turkey [14], the impacts of individual, familial, and parental factors on smoking outcomes among adolescents were assessed. In particular, we sought to examine the effects of parental smoking, alcohol consumption, and individual parenting behaviours. Whether the contribution of these factors differed between boys and girls was also examined.

## 2. Materials and Methods

### 2.1. Study Design and Population

This analysis is an extension of the cross-sectional study conducted in the Ankara province of Turkey in 2011 on *n* = 707 students (311 boys and 396 girls) and with an average age of 15.0 years. Depending on the prevalence obtained in the pilot study (45%), 95% confidence level, and 5% allowable error, the minimum sample size required was *n* = 414.Therefore, the study was sufficiently powered to detect meaningful differences. The participants were recruited randomly from schools in the Akyurt, Bala, and Çankaya districts using simple random sampling [14]. The study collected information on parental, student, and family demographics using a previously validated questionnaire. This study was approved by the Ankara Numune Research and Education Hospital Ethical Committee (Approval Number 2011-253) and the Governor’s Office of Ankara. All students and families provided written informed consent.

### 2.2. Smoking Consumption Outcome Variables

The Addictive Substances Attitudes Scale (ASAS) questionnaire was used to collect information on students’ smoking behaviour variables and attitudes towards smoking as well as alcohol. The questions included and used for the present study were “Have experienced smoking at least once until today”, “Was regularly smoking at least one cigarette daily”, and “The age of starting smoking” for students. Smoking tried at least once and regular smoking were coded as a dichotomous variable based on the responses to the first two questions. “The age of starting smoking” responses were based on multiple choice options <9 years, 10 years, 11 years, 12 years, 13 years, 14 years, and >15 years. For this outcome variable, the logistic regression was coded as <=12 years as an increase in risk.

### 2.3. Parental Behaviours Variables

The validated Parental Attitudes Scale (PAS) was used to assess the parental attitudes which were classified as democratic, protective/demanding, and authoritative. The responses were measured on a continuous scale.

### 2.4. Statistical Methods

Age was coded ≤15 years or >15 years to be consistent with other studies. Pairwise correlations were assessed between the parental attitudes. Parental attitudes variables were further categorised into quartiles. For the outcome variable “The age of starting smoking”, the categories were merged into ≤12 years and >12 years [15]. Based on the categorical nature of the outcome variables, a logistic regression was considered appropriate. A logistic regression model establishes a relationship between an outcome/dependent variable (binary) and a group of predictor/independent variables. These regression models use the logit-transformed probability to enable interpretation as a linear relationship to the independent variables. Here, for example, the binary outcome variable for “Have experienced smoking at least once until today” is indicating no/yes with (0/1) and p being the probability of y to be 1, *p* = P (Y = 1). The equation for the model:

logit(*p*) = log(*p*/(1 − *p*)) = β0 + β1* Age > 15 + β1* yes father smoking +… + βk*k

Here β0 is a constant and k is an independent variable. The odds ratio of the dependent variable is indicated through the exponential beta. From this odd ratio, the probability of the dependent variable is estimated. If the beta value (exponential) is above one, then the probability of a higher category increases, and if the beta is below one (exponential), then the probability of a higher category decreases. Exponential beta values are interpreted as a reference category, where the probability of the dependent variable increases or decreases.

To assess the contribution of each type of parental attitude of the mother or father, the 3 different parental attitudes (democratic, protective/demanding, and authoritative) were analysed in separate regression models. Unadjusted and adjusted models are presented, and results reported in terms of odds ratio and 95% confidence intervals. Data were further stratified by boys and girls to identify possible effect modification. Effect modification was used to assess whether the magnitude of the effect (odds ratio) of the predictor variables on smoking outcomes differs depending on the sex of the participants. *p* value less than 0.05 was considered as statistically significant. Statistical analyses were performed using Stata release 14.1 (Stata, College Station, TX, USA).

## 3. Results

A detailed description of the study participants is described elsewhere [14]. Briefly, Table 1 describes the parental, family, and student characteristics of the students (*n* = 707) according to their smoking behaviours. The majority (80.4%) were below the age of 15 years. Of these, 311 were boys with a mean age of 15.22 years (range 12 to 21, SD = 0.8) and 396 girls with a mean age of 15.0 years (range 13 to 18, SD = 0.5). A few students had both parents who were regular drinkers, 9 (1.2%), or smokers, 125 (17.6%). The education level of the parents differed, with more mothers (49.9%) having a primary education level compared to fathers (39.1%). In contrast, a greater number of fathers of the participants had a higher education (25.4%) compared to the mothers (16.2%). Nearly half the participants were from families with a good financial status 302 (42.7%) and less than 10% were from families with a poor financial status.

About 35.1% of females and 48.2% of males smoked (chi-square value =12.4, *p* value <0.001) and 19.6% of boys smoked in the last 30 days compared to 6.0% girls (chi-square value = 30.2, *p* value < 0.001). Boys were much younger (<9 years) when they first started smoking 31 (22.8%) [14]. Having experienced smoking at least once until today was reported more among students above 15 years (54.3%), having mother (68.0%) and/or father (68.7%) regularly use alcohol, and belonging to a very bad financial status (68.7%). Nearly half of the students with regular smoking have either one or both parent as a regular drinker.

Parental attitudes are summarised in Table S1 (online supplementary). Parents had higher democratic attitudes. Democratic scores of both the parents were higher for girls compared to boys, whereas protective demanding and authoritarian scores for parents were higher for boys. However, the parental attitudes were highly correlated irrespective of the gender.

Table 2 shows the results of fitting a logistic regression model to “Have experienced smoking at least once until today”. Being older and male increased odds of smoking at least once (OR = 1.8, 95% CI 1.2, 2.7, OR = 1.5, 95% CI 1.1, 2.1, respectively, from the adjusted model). Neither the smoking status of the father or mother was associated with this variable. But occasional alcohol use of both the mother and father increased odds (OR = 2.4, 95% CI 1.2, 4.7, OR = 1.5 95% CI 0.9, 2.3, respectively, from the adjusted model). Mothers’ democratic attitude was protective (OR = 0.5, 95% CI 0.3, 0.9) but not if protective and demanding (OR = 1.5, 95% CI 0.9, 2.4). Similarly, a democratic father was protective (OR = 0.6, 95% CI 0.3, 0.9). An authoritarian father (OR = 1.7, 95% CI 1.0, 2.7) increased odds of smoking at least once (Table 3).

**Figure 1:**
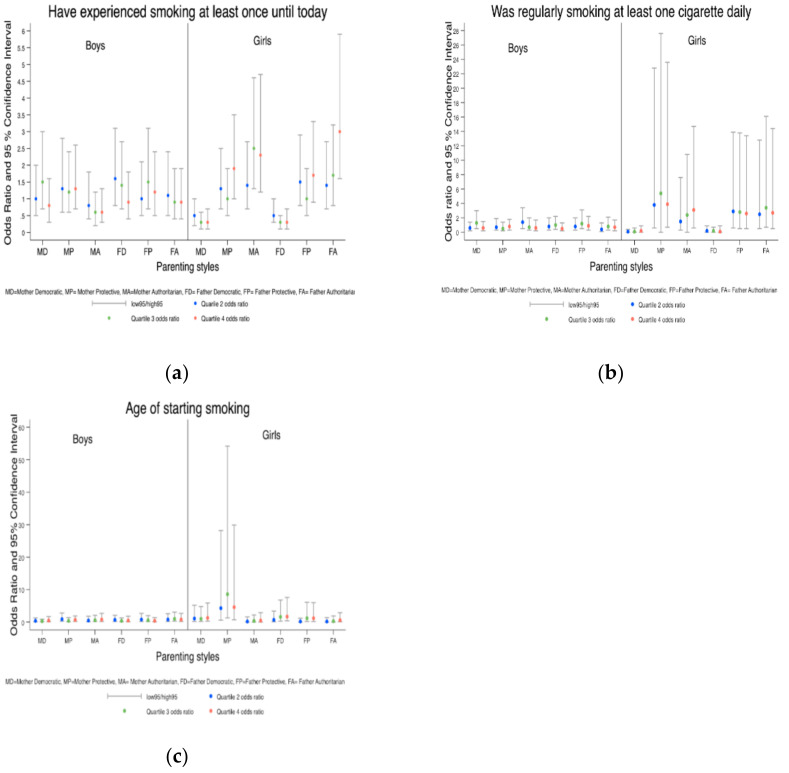
(**a**) Adjusted odds ratio of parental attitude variables for “Have experienced smoking at least once until today” by gender; (**b**) Adjusted odds ratio of parental attitude variables for “Was regularly smoking at least one cigarette daily” by gender; (**c**) Adjusted odds ratio of parental attitude variables for “Age of starting smoking” by gender.

When we stratified the analysis by boys and girls (Table 2), being older was only significant among girls (OR = 2.0, 95% CI 1.1, 3.7). Neither parent smoking was important among boys but fathers smoking increased odds for girls (OR = 1.5, 95% CI 1.0, 2.5). Occasional alcohol use of the father increased odds for boys. Conversely, occasional and regular alcohol use of the mother increased odds for girls but not boys. Mother and father democratic attitude was significantly protective for girls but for not boys (OR = 0.3, 95% CI 0.1, 0.7, OR = 0.3, 95% CI 0.1, 0.7, respectively, from the adjusted model). An authoritarian father and mother significantly increased the odds for only girls (Figure 1a).

Age more than 15 years and male increased odds of smoking regularly (OR = 2.8, 95% CI 1.6, 4.9, OR = 3.7, 95% CI 2.1, 6.6, respectively, from the adjusted model). Regular alcohol use of both mother and father increased the odds (OR = 5.2 95% CI 1.3, 19.6, OR = 4.4, 95% CI 1.4, 13.6, respectively). Education of father (OR = 0.1, 95% CI 0.0, 1.0), but not mother, was associated with this variable (Table 2). Both mother and father democratic attitude was protective (OR = 0.4, 95% CI 0.2, 1.0, OR = 0.4, 95% CI 0.1, 0.8, respectively, from the adjusted model) (Table 3).

When stratified by sex for regular smoking, being older was significant for boys (OR = 2.9, 95% CI 1.5, 5.6) and borderline significant for girls (OR = 2.6, 95% CI 0.8, 8.4). Education status of father and democratic attitude of both the parents (Table 2) were significantly protective for girls but not for boys.

Students having both parents who were regular drinkers or occasional drinkers increased the odds of smoking at least once (OR = 6.1, 95% CI 1.2, 29.8, OR = 2.6, 95% CI 1.4, 4.7, respectively, from the unadjusted model). However, regularly smoking students were more likely to have one or both parents as regular drinkers (OR = 4.7, 95% CI 1.5, 14.3, OR = 18.8, 95% CI 4.5, 77.6, respectively, from the unadjusted model). Students with one parent as a smoker increases the odds of smoking at least once (OR = 1.4, 95% CI 1.0, 1.9, unadjusted model) or smoking regularly (OR = 1.6, 95% CI 1.0, 2.7).

Table 2 shows the results of fitting a logistic regression model to “the age of starting smoking”. Being male increased the odds of smoking at an early age (adjusted OR = 3.2, 95% CI 1.7–5.8, from the adjusted model). The smoking status of the mother wasn’t significant. However, the smoking status of the father decreases the odds in the model (adjusted OR = 0.5, 95% CI 0.2, 0.9). None of the other variables were predictive.

When stratified by sex for the variable (Table 2), the smoking status of the mother was borderline significant for only boys (OR = 2.4, 95% CI 0.9, 6.6). Conversely, for girls, occasional use of alcohol by the father was significant. Financial status of the family was important in an unadjusted model but when adjusted for parental alcohol status, it became non-significant. A democratic mother decreased the odds for boys (OR = 0.3, 95% CI 0.1, 0.9), whereas a protective, demanding mother increased the odds for girls (OR = 8.6, 95% CI 1.3, 54.2) (Figure 1c).

## 4. Discussion

The burden of smoking among adolescents remains high, especially in countries such as Turkey where smoking cessation policies may be ineffective. The cost of smoking remains high with evidence reporting variations in household income and cigarette expenses among low-income and middle-income countries, i.e., 4.25% and 7.2% for low-income households and between 1.65% and 3% for the higher income households [16]. In addition to behavioural determinants, structural factors including legislation and regulation associated with tobacco use, tobacco industry advertising and lobbying, government revenue-raising (e.g., through taxes, levies, etc.) on tobacco sales, and health service capacity and funding also have an impact on tobacco experimentation and use by adolescents. The tobacco industry in Turkey continues to target adolescents with advertising despite legislations and regulations in the country [17]. Turkey has excise taxes on tobacco but does not necessarily discourage usage [16]. The health insurance system in Turkey doesn’t include smoking cessation services, especially tobacco dependence, further limiting the access [17]. A public health problem continues to emerge as the public health impacts and costs of ill health will be problematic long term.

Our study showed some interesting results. Firstly, both boys and girls were likely to start smoking at younger than 12 years of age. The smoking status of the mother increased the odds of boys starting smoking at a younger age but not girls. Regular alcohol consumption of the father impacted girls starting smoking younger but not boys. Both parents regularly drinking increased the odds of smoking. The effect of parental attitudes in boys compared to girls were not as consistent. Parental attitudes did not affect boys as much as girls, where the democratic attitude of parents was found to be protective. In boys, the democratic attitude of the mother seems to be responsible for decreasing the odds of smoking at an early age. Unexpectedly, neither income nor education was that important, suggesting that perhaps parental behaviours is the key, especially in school-aged children. This study adds to a growing body of literature that suggests parenting behaviours plays a major role in smoking among school-aged children.

Consistent with the previous study [9], our study identified that smoking at least once and regular smoking were higher in boys and in the older age group for both boys and girls. Here, 13.30% of students and 46.32% of boys started smoking before the age of 12 years. The significance of this point has been highlighted by Fidler et al. in their study, they demonstrated that at the age of 11, 14% of participants had at least once attempted to smoke and carried a higher risk of smoking for more than three years, compared to non-smokers with no history of attempted smoking. The phenomenon was termed by the researchers as a “sleeper effect” [18]. In contrast to a systematic review [19], we found adolescents were at a greater risk of smoking where one parent smoked rather than both. The effect was higher in magnitude where the smoking parent was the mother [20] and not the father. This may suggest cultural factors where still it is uncommon for acceptance of smoking among mothers. Other factors include parental approaches, perceived parental consent of smoking, and access to tobacco. The reduction of the parental smoking status as demonstrated by the odds ratio and the 95% confidence interval not excluding the null value, suggests chance or unexplained confounding on other smoking outcomes as possible explanations and this should not be ruled out.

Here, we showed that adolescents with parents who drink alcohol occasionally or regularly have a higher risk of smoking. To our knowledge, this is the first study to examine the impact of parental alcohol consumption on adolescent smoking in countries with similar demographics to that of the Republic of Turkey. It is unlikely to be a spurious finding as highlighted by a study that the mechanisms by which parents transmit substance use risk to children may be general, rather than substance specific [21], and also there is a proven common genetic propensity for alcohol and tobacco use [22]. In addition, boys are more impacted by paternal drinking habits compared to maternal drinking patterns. This highlights the role of fathers in the early childhood and adolescent phase of growth and the importance for parenting programs and other interventions to address risk-taking behaviours.

It is a complicated structure that adolescents start smoking and continue. In Turkey, like most other countries, parents don’t allow adolescents to smoke. Such an unapproved behaviour generally starts secretly without parents knowing. When parents become aware of it, sometime after the adolescent has begun smoking, only disapproving is not enough. Due to this, several interventions are needed to be planned in order to prevent adolescents from smoking. Turkey has been implementing action plans in tobacco control by following the regulations since 2008. Following all the precautions included in the M POWER (Monitor, Protect, Offer, Warn, Enforce, Raise) policy package, Turkey is recognized as one of the leading countries in tobacco control by the World Health Organization [17]. However, smoking rates are still relatively high in the country (almost half of the males who are older than 15 years of age smoke) and it remains one of the most common health problems. Decreasing smoking rates in society, especially in parents, is likely to decrease smoking rates in adolescents. Preventing smoking is included in the school curriculum and most primary education providers organize awareness events at least once a year. For most adolescents, smoking remains a symbol of pop-culture and is taken as a part of their transition into adulthood. Our findings showed that parental democratic parenting styles were protective. Adolescent behaviour is closely dependent on the relationship between the child and parents in Turkish culture which sets expectations and encourages children “to be a good child” and behave accordingly [23]. Prevention could target early parenting styles where authoritarian and protective parenting styles are common in countries such as Turkey. Early cultural and family functioning focused interventions could improve the parent and child relationships to reduce the harms of smoking uptake. Brief and theory-based family interventions aimed at parenting styles are likely to have a long-term preventive effect on adolescents’ tobacco usage. In light of our findings, it is strongly suggested that tobacco prevention programs targeting parent–adolescent communication be developed, i.e., a model development of the child where the parent, in an accepting and steady manner, fosters a climate to ensure cooperation, fairness, equality, and mutual respect. Further, the effectiveness of these interventions should be assessed regularly by monitoring the prevalence of tobacco use among the adolescents [17].

It seems that there is an assumption that family income or affluence [24], or parental education [25], are important, but that is not what we found here, possibly because the variables we included are crude markers of these effects or that peers at this age group are far more important. However, our results indicate that girls are less likely to smoke at an early age if they belong to an affluent family [26]. Further, in the present study, parental education or family income were considered as a measure of adolescent socio-economic status. Future studies with a multivariate model having both adolescent and parental education and financial status should be included to assess whether both socio-economic status as an independent measure of familial and environmental factors may contribute to updated or continued smoking in adolescents [27]. Parents of low-income families may exhibit stress and anxiety, and this may contribute to the risk-taking behaviours of adolescents. More studies are needed to assess the mental health well-being of parents, family income, and their combined impacts on smoking behaviours among adolescents.

A child’s likelihood of smoking is impacted by parenting practices both regarding smoking and more generally. Parental smoking behaviour seems to be less important than parental attitude towards smoking. Supporting the previous studies [28,29], children whose parents are democratic or authoritative (high support and control) are less likely to become smokers. On the other hand, the authoritarian parenting style results in more smoking among adolescents [30]. Our findings are in concordance with a previous study [31] where high parental control contributed to girls’ uncommon risky behaviours. This could be due to the higher level of emotional development amongst the female adolescents. For preventive interventions, smoking-specific parenting practices should be considered as they were unique predictors of adolescent smoking. Furthermore, smoking-related parenting practices appears to be easily influenceable compared to overall parenting styles, and hence, should be targeted for interventions.

This study is the first of its kind, with no previous work which considered the effects of fathers’ and mothers’ parenting behaviours separately and whether these effects remain with alcohol consumption and smoking behaviours included in the models. A strength of this study is the large sample size, allowing enough power to assess differences by boys and girls. There are limitations that need to be considered when interpreting the findings. The data source for the study has been limited to one district, Ankara. Thus, results need to be generalised prudently as characteristics of the district included differ from those of other regions in Turkey. The design of this study being cross-sectional, therefore, the school may not be representative of the general population although the smoking habits have not substantially declined since these data were collected. Moreover, all data are self-reported, so there may be chances of recall or social-desirability bias and underreporting. Some of the variables were crude and may have contributed to effects to the null such as the family financial status. Another important limitation to be considered is that all the factors analysed in the present study are external and it is imperative to understand and include those factors that influence smoking through internal mechanisms. Nevertheless, this study has addressed several gaps and contributed to limited studies on the literature on adolescent smoking behaviour and mothers’ and fathers’ parenting styles from countries in Europe that may be different to other regions in the world.

## 5. Conclusions

In summary, our study shows that individual parental styles and behaviours elevate the risk of adolescent smoking and starting at an early age. In addition, alcohol consumption by either parent also elevated the risk but fathers smoking increased the risk among girls. Parental alcohol consumption and smoking behaviours are also important and should be addressed.

## Figures and Tables

**Table 1:** Parental, family and student characteristics according to smoking behaviour of the students.

Characteristics	*N* (%)	Have Experienced Smoking at Least Once until Today, *N* (%)	Was Regularly Smoking at Least One Cigarette Daily, *N* (%)
**Age, *n* (%)**			
≤15	569(80.4%)	214 (37.6%)	51 (8.9%)
>15	138 (19.5%)	75 (54.3%)	34 (24.6%)
**Sex**			
Males	311 (43.9%)	150 (48.2%)	61 (19.6%)
Females	396 (56.0%)	139 (35.1%)	24 (6.0%)
**Father Smoking**			
No	360 (50.9%)	132 (36.6%)	36 (10.0%)
Yes	347 (49.0%)	157 (45.2%)	49 (14.1%)
**Mother Smoking**			
No	528 (74.6%)	210 (39.7%)	63 (11.9%)
Yes	179 (25.3%)	79 (44.1%)	22 (12.2%)
**Father Alcohol Use**			
Don’t drink	533 (75.3%)	196 (36.7%)	54 (10.1%)
Occasional	149 (21.0%)	76 (51.0%)	19 (12.7%)
Regular	25 (3.5%)	17 (68.0%)	12 (48.0%)
**Mother Alcohol Use**			
Don’t drink	628 (88.8%)	239 (38.0%)	66 (10.5%)
Occasional	63 (8.9%)	39 (61.9%)	11 (17.4%)
Regular	16 (2.2%)	11 (68.7%)	8 (50.0%)
**Mother Education**			
Illiterate	24 (3.3%)	12 (50.0%)	4 (16.6%)
Primary School Graduate	353 (49.9%)	144 (40.7%)	41 (11.6%)
Secondary school graduate	215 (30.4%)	86 (40.0%)	25 (11.6%)
Higher education	115 (16.2%)	47 (40.8%)	15 (13.0%)
**Father Education**			
Illiterate	14 (1.9%)	7 (50.0%)	5 (35.7%)
Primary School Graduate	277 (39.1%)	114 (41.1%)	29 (10.4%)
Secondary school graduate	236 (33.3%)	92 (38.9%)	55 (10.5%)
Higher education	180 (25.4%)	76 (42.2%)	26 (14.4%)
**Financial Status**			
Very poor	16 (2.2%)	11 (68.7%)	5 (31.2%)
Poor	43 (6.0%)	18 (41.8%)	5 (11.6%)
More or less same	278 (39.3%)	111 (39.9%)	28 (10.0%)
Good	302 (42.7%)	113 (37.4%)	32 (10.6%)
Extremely good	68 (9.6%)	36 (52.9%)	15 (22.0%)

Father Smoking and Mother Smoking represents current smoking status.

**Table 2:** Adjusted odds ratio for smoking behaviour outcomes.

Explanatory Variables	Have Experienced Smoking at Least Once until Today Adjusted OR (95% CI)			Was Regularly Smoking at Least One Cigarette Daily Adjusted OR (95% CI)			Age of Starting Smoking Adjusted OR (95% CI)		
	All	Boys	Girls	All	Boys	Girls	All	Boys	Girls
**Age, >15**	1.8 (1.2–2.7) *	1.6 (0.9–2.8) ^#^	2.0 (1.1–3.7) *	2.8 (1.6–4.9) **	2.9 (1.5–5.6) *	2.6 (0.8–8.4) ^#^	–	–	–
**Father Smoking,**									
Yes	1.3 (0.9–1.8)	1.1 (0.7–1.9)	1.5 (1.0–2.5) *	1.3 (0.7–2.2)	1.8 (0.9–3.6) ^#^	0.5 (0.1–1.5)	0.5 (0.2–0.9) *	0.6 (0.2–1.4)	0.5 (0.1–1.5)
**Mother Smoking,**									
Yes	0.8 (0.6–1.3)	0.8 (0.4–1.5)	0.99 (0.5–1.6)	0.68 (0.3–1.2)	0.5 (0.2–1.3)	0.7 (0.2–2.4)	1.4 (0.7–2.8)	2.4 (0.9–6.6) ^#^	0.4 (0.1–1.5)
**Father Alcohol Use**									
Occasional	1.5 (0.9–2.3) ^#^	2.3 (1.2–4.7) *	1.1 (0.5–2.0)	1.2 (0.6–2.5)	0.9 (0.4–2.2)	3.1 (0.9–10.5) ^#^	1.4 (0.6–3.2)	0.6 (0.2–1.8)	5.0 (1.1–23.1) *
Regular	1.9 (0.7–5.2)	3.0 (0.7–11.7)	0.7 (0.1–4.4)	4.4 (1.4–13.6) *	4.8 (1.2–19.1) *	3.5 (0.2–45.5)	2.4 (0.5–10.1)	2.8 (0.–16.82)	1.0 (0.0–57.0)
**Mother Alcohol Use**									
Occasional	2.4 (1.2–4.7) *	1.8 (0.7–4.3)	3.1 (1.1–8.8) *	1.5 (0.6–3.8)	2.4 (0.8–7.1)	0.3 (0.0–4.0)	0.5 (0.2–1.6)	0.5 (0.1–2.1)	0.6 (0.0–5.6)
Regular	2.3 (0.7–8.1)	0.9 (0.1–7.3)	10.5(1.2–88.0) *	5.2 (1.3–19.) *	7.7 (0.8–67.7) ^#^	7.0 (0.7–70.3) ^#^	1.9 (0.3–12.4)	–	1.9 (0.0–59.7)
**Education of Mother**									
Primary	0.8 (0.3–2.2)	0.7 (0.1–4.2)	1.14 (0.–4.31)	3.4 (0.4–25.)	2.0 (0.1–24.8)	4.0 (0.1–111.7)	1.0 (0.1–9.4)	0.8 (0.0–12.3)	
Secondary	0.7 (0.2–1.9)	0.3 (0.0–1.9)	1.54 (0.3–6.2)	2.5 (0.3–18.7)	1.3 (0.1–17.1)	4.0 (0.1–116.5)	0.4 (0.0–3.7)	0.5 (0.0–9.2)	–
Higher	0.4 (0.1–1.4)	0.2 (0.0–1.4)	1.06 (0.2–5.0)	1.48 (0.1–12.1)	0.57 (0.0–8.6)	5.4 (0.1–173.7)	0.4 (0.0–4.9)	0.4 (0.0–9.0)	
**Education of Father**									
Primary	0.9 (0.2–3.4)	1.2 (0.1–11.1)	0.81 (0.1–4.7)	0.09 (0.0–0.5) *	0.2 (0.0–2.8)	0.0 (0.0–0.4) *	0.0 (0.0–1.6)	0.7 (0.2–2.6)	0.0 (0.0–3.6)
Secondary	0.9 (0.2–3.6)	2.2 (0.2–20.4)	0.58 (0.0–3.4)	0.1 (0.0–0.8) *	0.2 (0.0–3.4)	0.0 (0.0–0.6) *	0.1 (0.0–1.9)	0.6 (0.2–1.8)	0.0 (0.0–6.5)
Higher	1.0 (0.2–4.1)	2.3 (0.2–22.4)	0.6 (0.1–4.0)	0.1 (0.0–1.0) ^#^	0.4 (0.0–5.5)	0.0 (0.0–0.5) *	0.1 (0.0–2.5)	–	0.0 (0.0–5.8)
**Financial Status**									
Poor	0.3 (0.1–1.4)	0.2 (0.0–2.7)	0.4 (0.0–2.5)	0.3 (0.0–2.2)	0.9 (0.0–11.5)	0.0 (0.0–1.7)	0.2 (0.0–1.6)	1.3 (0.1–17.0)	–
More or less same	0.3 (0.1–1.1) ^#^	0.1 (0.0–1.1) ^#^	0.7 (0.1–3.6)	0.3 (0.0–1.6)	0.6 (0.0–6.0)	0.1 (0.0–1.8)	0.4 (0.0–2.6)	2.0 (0.2–19.1)	0.1 (0.0–1.2) ^#^
Good	0.3 (0.1–1.1) ^#^	0.1 (0.0–1.4) ^#^	0.6 (0.1–2.8)	0.4 (0.1–2.1)	0.8 (0.0–8.3)	0.1 (0.0–1.7)	0.3 (0.0–2.1)	1.2 (0.1–12.5)	0.2 (0.0–1.) ^#^
Extremely good	0.6 (0.1–2.3)	0.3 (0.0–3.5)	1.0 (0.1–5.8)	1.1 (0.2–5.9)	1.8 (0.1–20.2)	0.8 (0.0–9.6)	0.7 (0.1–5.5)	2.9 (0.2–33.0)	–

Age < 15 years, no category for father smoking status, no for mother smoking status, no use of alcohol by mother, no use of alcohol by father, illiterate category for mother education status, illiterate category for father education status, and very bad financial status were used as reference categories and were coded as 0. ** *p* < 0.001, * *p* < 0.05, ^#^ *p* < 0.09.

**Table 3:** Adjusted odds ratio of parental attitude variables for smoking behaviour outcomes.

Explanatory Variables Parenting Attitude	Have Experienced Smoking at Least Once until Today Adjusted OR (95% CI)	Was Regularly Smoking at Least One Cigarette Daily Adjusted OR (95% CI)	Age of Starting Smoking Adjusted OR (95% CI)
	All	All	All
**Mother Democratic**			
Quartile 2	0.7 (0.5–1.2)	0.4 (0.2–0.8) *	0.6 (0.3–1.4)
Quartile 3	0.6 (0.4–1.0) ^#^	0.7 (0.3–1.4)	0.5 (0.2–1.2)
Quartile 4	0.5 (0.3–0.9) *	0.4 (0.2–1.0) ^#^	0.8 (0.3–1.9)
**Protective Demanding**			
Quartile 2	1.3 (0.8–2.1)	1.09 (0.5–2.3)	1.3 (0.5–2.9)
Quartile 3	1.2 (0.7–1.8)	1.08 (0.5–2.1)	0.8 (0.3–1.8)
Quartile 4	1.5 (0.9–2.4) ^#^	1.14 (0.5–2.2)	0.9 (0.4–2.1)
**Authoritarian**			
Quartile 2	1.1 (0.7–1.8)	1.5 (0.7–3.2)	0.7 (0.3–1.5)
Quartile 3	1.3 (0.8–2.1)	1.1 (0.5–2.4)	0.8 (0.4–1.7)
Quartile 4	1.2 (0.8–2.0)	1.1 (0.5–2.5)	1.1 (0.5–2.4)
**Father**			
Democratic			
Quartile 2	0.9 (0.5–1.4)	0.6 (0.3–1.2)	0.8 (0.3–1.7)
Quartile 3	0.6 (0.4–0.9) *	0.6 (0.3–1.2)	0.8 (0.3–1.8)
Quartile 4	0.6 (0.3–0.9) *	0.4 (0.1–0.8) *	0.9 (0.4–2.2)
**Protective Demanding**			
Quartile 2	1.3 (0.8–2.0)	1.2 (0.5–2.5)	0.5 (0.2–1.3)
Quartile 3	1.2 (0.8–2.0)	1.4 (0.6–3.0)	0.6 (0.2–1.5)
Quartile 4	1.3 (0.8–2.1)	1.2 (0.5–2.6)	0.6 (0.2–1.4)
**Authoritarian**			
Quartile 2	1.3 (0.8–2.0)	0.7 (0.3–1.5)	0.5 (0.2–1.2)
Quartile 3	1.3 (0.8–2.1)	1.2 (0.6–2.5)	0.7(0.3–1.6)
Quartile 4	1.7 (1.0–2.7) *	1.0 (0.5–2.2)	0.7 (0.3–1.8)

* *p* < 0.05, ^#^ *p* < 0.09.

## Data Availability

The data presented in this study are available on request from the corresponding author. The data are not publicly available due to the Turkish law.

## Supplementary Materials

The following are available online at https://www.mdpi.com/article/10.3390/ijerph18073740/s1, Table S1: Correlation of parental attitudes according to gender of the students.
