# Supplementary material for: Impact of Individual, Familial and Parental Factors on Adolescent Smoking in Turkey
Source: Int J Environ Res Public Health. 2021 Apr 2;18(7):3740. doi: 10.3390/ijerph18073740 (PMC8038305; doi:10.3390/ijerph18073740)
Supplement: Supplementary file 1 [file ijerph-18-03740-s001.pdf]

Table S1: Parental attitudes according to gender of the students.

Gender	Mother Parental attitudes<br>Mean ± SD	Father parental attitudes<br>Mean ± SD		
**Boys**		**Democratic**<br>48.33 ± 11.03	**Protective Demanding**<br>41.81 ± 12.02	**Authoritarian**<br>25.72 ± 9.03
	**Democratic**<br>49.09 ± 11.04	0.82*, 0.00	0.46*, 0.00	0.28*, 0.00
	**Protective Demanding**<br>42.29 ± 11.98	0.52*, 0.00	0.91*, 0.00	0.77*, 0.00
	**Authoritarian**<br>25.25 ± 9.24	0.35*, 0.00	0.81*, 0.00	0.91*, 0.00
**Girls**		**Democratic**<br>49.09 ± 10.32	**Protective Demanding**<br>39.92 ± 11.25	**Authoritarian**<br>23.54 ± 8.75
	**Democratic**<br>50.03 ± 10.62	0.83*, 0.00	0.19*, 0.00	-0.02, 0.57
	**Protective Demanding**<br>40.68 ± 10.97	0.23*, 0.00	0.89*, 0.00	0.78*, 0.00
	**Authoritarian**<br>23.16 ± 8.95	0.02, 0.65	0.75*, 0.00	0.91*, 0.00

* $p < 0.05$
